# Determination of the beam quality correction factor $k_{Q_{\mathrm{msr}}}$ for the PTW Semiflex 3D ionization chamber for the reference dosimetry at ZAP‐X

**DOI:** 10.1002/acm2.14610

**Published:** 2025-01-07

**Authors:** Katrin Saße, Karina Albers, Daniela Eulenstein, Georg Weidlich, Björn Poppe, Hui Khee Looe

**Affiliations:** ^1^ University Clinic for Medical Radiation Physics Medical Campus Pius Hospital Carl von Ossietzky University Oldenburg Germany; ^2^ Zap Surgical Medical Physics Department PTW‐Freiburg Freiburg Germany; ^3^ ZAP Surgical San Carlos California USA

**Keywords:** beam correction factor, small field, ZAP‐X

## Abstract

**Purpose:**

The self‐shielding radiosurgery system ZAP‐X consists of a 3 MV linear accelerator and eight round collimators. For this system, it is a common practice to perform the reference dosimetry using the largest 25 mm diameter collimator at a source‐to‐axis distance (SAD) of 45 cm with the PTW Semiflex3D chamber placed at a measurement depth of 7 mm in water. Existing dosimetry protocols do not provide correction for these measurement conditions. Therefore, Monte Carlo simulations were performed to quantify the associated beam quality correction factor $k_{Q_{\mathrm{msr}},Q_{\mathrm{ref}}}^{f_{\mathrm{msr}},f_{\mathrm{ref}}}$.

**Methods:**

The $k_{Q_{\mathrm{msr}},Q_{\mathrm{ref}}}^{f_{\mathrm{msr}},f_{\mathrm{ref}}}$ of the Semiflex3D chamber was computed from the ratio of the absorbed doses in a water voxel and in the sensitive air volume of the chamber simulated using a ^60^Co spectrum as the calibration beam quality (*Q*_ref_) and the spectrum of the ZAP‐X 3 MV photon beam (*Q*_msr_). $k_{Q_{\mathrm{msr}},Q_{\mathrm{ref}}}^{f_{\mathrm{msr}},f_{\mathrm{ref}}}$ was computed as a function of measurement depth from 4 to 50 mm. Furthermore, detailed simulations were performed to determine the individual chamber's perturbation correction factors by modifying the chamber's model step‐wise.

**Results:**

All perturbation correction factors, except *S*_w,air_ ⋅*P*_fl_, show depth‐dependent behavior up to a depth of 15 mm. In particular, the volume‐averaging *P*_vol_ and density *P*_dens_ perturbation correction factors and, consequently, the resulting gradient perturbation correction factor *P*_gr _= *P*_vol_∙*P*_dens_ increase with decreasing measurement depth. Therefore, $k_{Q_{\mathrm{msr}},Q_{\mathrm{ref}}}^{f_{\mathrm{msr}},f_{\mathrm{ref}}}$ is larger than unity, amounting to 1.0104±0.0072 at 7 mm measurement depth. At larger depths (> 15 mm), the $k_{Q_{\mathrm{msr}},Q_{\mathrm{ref}}}^{f_{\mathrm{msr}},f_{\mathrm{ref}}}=0.9964\;\pm \;0.0025$ can be considered as constant.

**Conclusion:**

At small measurement depths, $k_{Q_{\mathrm{msr}},Q_{\mathrm{ref}}}^{f_{\mathrm{msr}},f_{\mathrm{ref}}}$ was found to be depth‐dependent with values larger than unity due to the gradient‐related perturbation factors. Therefore, the uncertainty related to the chamber's positioning can be reduced by performing the reference dosimetry at ZAP‐X at depths larger than 15 mm, where $k_{Q_{\mathrm{msr}},Q_{\mathrm{ref}}}^{f_{\mathrm{msr}},f_{\mathrm{ref}}}$ can be regarded as depth independent.

## 1 INTRODUCTION

National and international dosimetry protocols^1^, ^2^, ^3^ provide guidelines to perform reference dosimetry for megavoltage photon beams using a calibrated ionization chamber. Since ^60^Co is usually used as the calibration beam quality for these chambers, a beam quality correction factor $k_{Q_{\mathrm{msr}},Q_{\mathrm{ref}}}^{f_{\mathrm{msr}},f_{\mathrm{ref}}}$ for the machine‐specific reference (msr) conditions (TRS 483) is applied to account for the difference in the chamber's dose response when irradiated with a high‐energy photon beam from a clinical linear accelerator (linac). These factors are commonly determined by employing Monte Carlo simulations using detailed chamber models and photon spectral fluence distributions or beam qualities. The results, that is, the chamber‐specific correction factors as a function of beam quality, are compiled and provided by the dosimetry protocols for commonly used chambers. The clinical beam quality is characterized by a single parameter, the beam quality index *Q*. According to the international TRS 398^4^ and the German DIN 6800–2 protocols, *Q* is defined by the TPR_20,10_, which represents the ratio of the dose at 20 cm and 10 cm water depths using a fixed source‐to‐detector (SDD) distance of 100 cm for a field size of 10 cm x 10 cm. The AAPM TG‐51 protocol adopts the percent depth dose (PDD)(10 cm)_x_ method as the beam quality index, which represents the percentage depth dose value at 10 cm without the contribution from electron contaminations. Reference dosimetry at C‐arm linacs is then commonly performed using a reference field size of 10 cm x 10 cm in 10 cm depth in water at either an SSD of 100 cm or a source‐to‐axis distance (SAD) of 100 cm.

The conditions for determining the beam quality index Q and reference dosimetry described above cannot always be achieved, such as at Tomotherapy, GammaKnife, or Cyberknife, either due to field size or distance limitations.^1^, ^5^ For these machines, dedicated simulations of the required $k_{Q_{\mathrm{msr}},Q_{\mathrm{ref}}}^{f_{\mathrm{msr}},f_{\mathrm{ref}}}$ have been performed for the corresponding msr conditions, under which the reference dosimetry is usually performed. Recently a novel self‐shielding radiosurgery system, the ZAP‐X (Zap Surgical Systems, Inc. of San Carlos, California), became available.^6^, ^7^ The nominal photon beam energy of the ZAP‐X system is 3 MV. The radiation fields are collimated using cylindrical cones, producing field sizes with diameters between 4 and 25 mm at an SAD of 45 cm.

The calibration of the machine output, that is, the reference dosimetry, is performed according to the manufacturer's recommendations using the largest cone diameter of 25 mm at an SSD of 45 cm. The most commonly used chamber for this purpose is the Semiflex 3D chamber (type 31021, PTW Freiburg, Germany) positioned at a depth of dose maximum, *d*_max_, of 7 mm or at 5 cm depth. To the best of the authors’ knowledge, no beam quality correction factor exists for this chamber yet under these measurement conditions.

The aim of this paper are (i) to calculate the $k_{Q_{\mathrm{msr}},Q_{\mathrm{ref}}}^{f_{\mathrm{msr}},f_{\mathrm{ref}}}$ factor using Monte Carlo simulations for the Semiflex 3D chamber to be used in reference dosimetry of the ZAP‐X, (ii) to study the depth dependence of the $k_{Q_{\mathrm{msr}},Q_{\mathrm{ref}}}^{f_{\mathrm{msr}},f_{\mathrm{ref}}}$ factor, and (iii) to quantify the individual chamber's perturbation correction factors. Thereby, based on the results derived from this comprehensive study, a dedicated protocol for reference dosimetry at ZAP‐X using the Semiflex 3D chamber can be proposed.

## 2 MATERIAL AND METHODS

### 2.1 The ZAP‐X system

The ZAP‐X system is a vault free radiosurgery system for treating small brain lesions. The system comprises a 3 MV linear accelerator and eight collimators with diameters between 4 and 25 mm. Due to the limited space in the shielding sphere,^8^ the SAD is 45 cm. The machine has two rotation axes: a co‐planar rotation axis around the patient cranial‐caudal axis and an additional oblique axis tilted by 45°. By combining these two gantry axes, a steradian of nearly 2π radiation can be achieved. A kV‐imaging system integrated inside the self‐shielding sphere is used to guide patient positioning.

### 2.2 The Semiflex 3D chamber

The Semiflex 3D chamber (type 31021, PTW Freiburg, Germany), an air‐filled ionization chamber, was investigated. The sensitive volume has a radius of 2.4 mm and is 4.8 mm long, resulting in a volume of 0.07 cm^3^ suitable for reference dosimetry in field dimensions down to 25 mm.

### 2.3 Formulism for reference dosimetry

The absorbed dose‐to‐water $D_{w,Q_{\mathrm{msr}}}^{f_{\mathrm{msr}}}$ under the msr condition with beam quality *Q*_msr_ can be obtained as

(1) $$D_{w,Q_{\mathrm{msr}}}^{f_{\mathrm{msr}}}=\left(M_{Q_{\mathrm{msr}}}^{f_{\mathrm{msr}}}-M_{0}\right)\;\cdot N_{D_{w},Q_{\mathrm{ref}}}^{f_{\mathrm{ref}}}\cdot k_{Q_{\mathrm{msr}},Q_{\mathrm{ref}}}^{f_{\mathrm{msr}},f_{\mathrm{ref}}}$$

where $\left(M_{Q_{\mathrm{msr}}}^{f_{\mathrm{msr}}}-M_{0}\right)$ is the background‐subtracted measured signal, corrected for the signal influencing factors, such as air density, ion recombination, polarity effect, and so forth. $N_{D_{w},Q_{\mathrm{ref}}}^{f_{\mathrm{ref}}}$ is the chamber‐specific calibration coefficient obtained using a ^60^Co source (beam quality *Q*_ref_). The change in the chamber's dose response attributed to the different beam qualities (*Q*_ref_ and *Q*_msr_) is accounted for by the beam quality correction factor $k_{Q_{\mathrm{msr}},Q_{\mathrm{ref}}}^{f_{\mathrm{msr}},f_{\mathrm{ref}}}$.

The beam quality correction factor $k_{Q_{\mathrm{msr}},Q_{\mathrm{ref}}}^{f_{\mathrm{msr}},f_{\mathrm{ref}}}$ at the measurement depth *z* can be expressed as

(2) $$k_{Q_{\mathrm{msr}},Q_{\mathrm{ref}}}^{f_{\mathrm{msr}},f_{\mathrm{ref}}}\left(z\right)=\frac{f_{Q_{\mathrm{msr}}}\left(z\right)}{f_{Q_{\mathrm{ref}}}}=\frac{{\left(\frac{D_{w}\left(z\right)}{D_{\mathrm{IC}}\left(z\right)}\right)}_{Q_{\mathrm{msr}}}^{f_{\mathrm{msr}}}}{{\left(\frac{D_{w}}{D_{\mathrm{IC}}}\right)}_{Q_{\mathrm{ref}}}^{f_{\mathrm{ref}}}}\;$$

that represents the ratio of the *f_Q_* factors for the beam qualities $Q_{\mathrm{msr}}$ and $Q_{\mathrm{ref}}$. These *f_Q_* factors were determined from the ratio of the absorbed dose‐to‐water *D*_W_ and absorbed dose‐to‐air in the sensitive air volume of the ionization chamber *D*_IC._ In this work, the factor $k_{Q_{\mathrm{msr}},Q_{\mathrm{ref}}}^{f_{\mathrm{msr}},f_{\mathrm{ref}}}\left(z\right)$ as indicated in equation 2 has been investigated as a function of the measurement depth (*z*).

### 2.4 Monte Carlo simulations

The simulations were performed using the EGSnrc 2019a package and the user code *egs_chamber*.^9^, ^10^ The Semiflex 3D chamber was modeled according to the construction drawing provided by the manufacturer. The chamber was positioned with its axis perpendicular to the beam's axis (radial). The measurement depth (*z*) indicates the depth of the chamber's reference point. The corresponding absorbed dose‐to‐water was scored by replacing the chamber model with a 0.2 mm x 0.2 mm x 0.2 mm water voxel.

For the simulations with *Q*_ref_, the chamber was positioned at 5 cm depth in water with an SSD of 95 cm. The absorbed dose‐to‐water was scored under the same conditions. A collimated point source with a ^60^Co spectrum, as provided in the EGSnrc package, was implemented, producing a field size of 10 cm x 10 cm at the measurement depth. Similarly, a collimated point source was implemented using the photon spectrum of the 3 MV photon beam as provided by the manufacturer for the simulation with *Q*_msr._ The beam was collimated to a diameter of 25 mm at the surface of the phantom at an SSD of 45 cm, corresponding to the largest available cone. The measurement depth was varied between 4–50 mm according to equation 2.

All simulation parameters used in this study are provided in Table 1.

**TABLE 1:** Simulation settings in EGSnrc.

Item name	Description
Code	EGSnrc/egs_chamber
ECUT	512 keV
PCUT	10 keV
**Variance reduction techniques**	
Photon‐cross‐ section‐enhancement (XCSE)	XCSE factor = 512
Russian Roulette	Rejection factor = 521, E < 512 keV
Intermedia phase‐space‐scoring (IPSS)	Active
# histories / statistical uncertainty	100 single batches with 1 × 10^10^ histories each Results were computed from the mean values and the associated uncertainty as standard error of mean.

### 2.5 Chamber's perturbation correction factors

The individual chamber's perturbation correction factors $P_{i}$ and the water‐to‐air stopping power ratio $S_{w,\mathrm{air}}$ have been investigated as a function of measurement depth according to equation 3:

(3) $$f_{Q_{\mathrm{msr}}}\left(z\right)=S_{w,\mathrm{air}}\;\left(z\right)\cdot \prod P_{i}\left(z\right)$$

**FIGURE 1:**
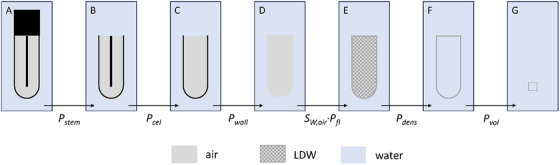
Stepwise modification of the model of the Semiflex 3D chamber (not to scale) to quantify the individual chamber's perturbation correction factors *P*_i_ according to Tekin et al.^11^ and Bouchard et al.^12^; LDW with a density of 1.225 mg cm^3^. LDW, low‐density water.

Thereby, the individual perturbation correction factors *P*_stem_, *P*_cel_ *,P*_wall_, $S_{w,\mathrm{air}}\cdot P_{\mathrm{fl}}$, *P*_dens_ and *P*_vol_ were quantified by modifying the chamber's model stepwise in the simulations as performed in.^11^ Each perturbation correction factor was computed as the ratio of the energy deposited in the scoring volume between two subsequent steps in Figure 1.

Firstly, the complete chamber was simulated in (A). In the next three steps, the chamber stem (B), central electrode (C) and chamber wall (D) were replaced one after the other with water, to obtain *P*_stem_, *P*_cel_ and *P*_wall_, respectively. To determine $S_{w,\mathrm{air}}\cdot P_{\mathrm{fl}}$, the air volume was replaced in step (E) with low‐density water (LDW), that is, water with the density of air (1.225 mg cm^3^). Consequently, the $P_{\mathrm{dens}}$ was determined by replacing LDW with water of normal density (F). In the last step, the volume‐averaging effect *P*_vol_ represents the ratio between the energy deposited in water with the volume of the sensitive volume of the chamber and that in a small water voxel (0.2 mm x 0.2 mm x 0.2 mm). The combined effect between steps (E) and (G) is also referred to as the gradient effect *P*_gr_ =$\;P_{\mathrm{dens}}\cdot$ *P*_vol._

## 3 RESULTS

### 3.1 Beam quality correction factors $k_{Q_{\mathrm{msr}},Q_{\mathrm{ref}}}^{f_{\mathrm{msr}},f_{\mathrm{ref}}}$

**FIGURE 2:**
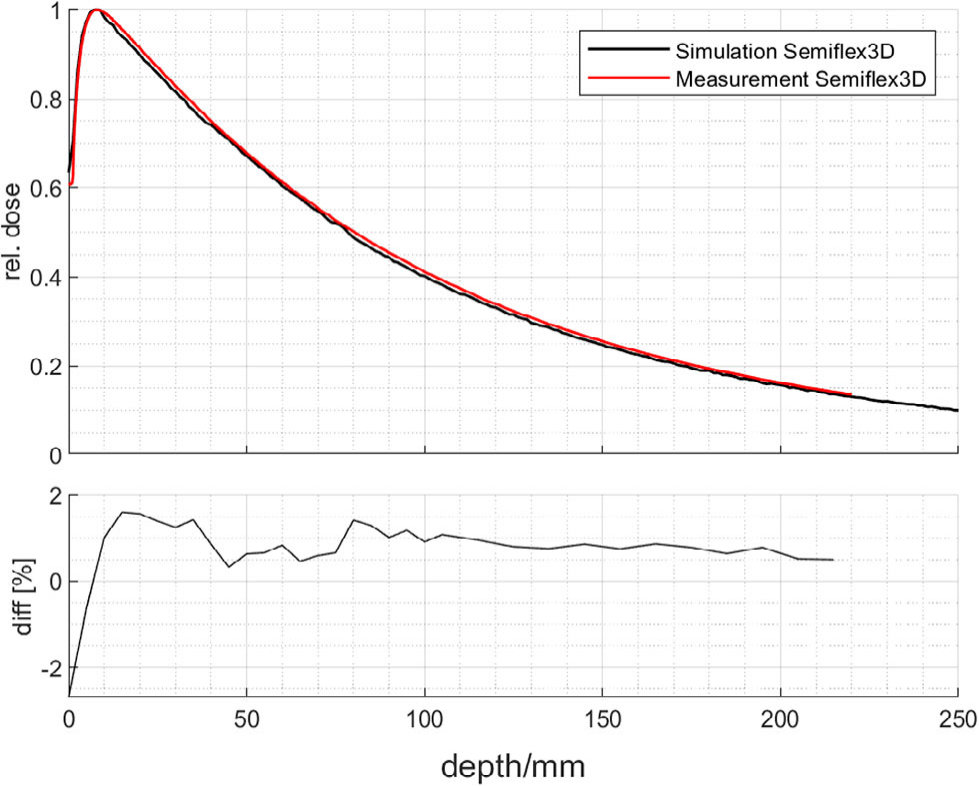
Comparison of the simulated (black line) and measured (red line) PDD of the Semiflex3D using the largest 25 mm diameter cone.

Firstly, the simulated and the measured depth dose curves of the 25 mm cone obtained using the Semiflex 3D chamber were compared in Figure 2. The deviation between the simulation and measurement, normalized to the maximum dose, is less than 1.6% over the entire range, indicating the validity of the 3 MV spectrum of the ZAP‐X machine supplied by the manufacturer.

**FIGURE 3:**
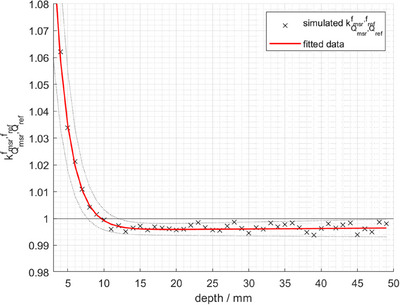
Simulated $k_{Q_{msr},Q_{ref}}^{f_{msr},f_{ref}}\left(z\right)$ (black crosses) of the Semiflex 3D ionization chamber as a function of measurement depth *z*. The data was fitted according to equation 4 (red line).

The simulated $f_{Q_{\mathrm{ref}}}$ for the calibration condition amounts to 1.0967 ± 0.0004. The depth‐dependent factors $k_{Q_{\mathrm{msr}},Q_{\mathrm{ref}}}^{f_{\mathrm{msr}},f_{\mathrm{ref}}}\left(z\right)$, calculated as the ratio $f_{Q_{\mathrm{msr}}}\left(z\right)/f_{Q_{\mathrm{ref}}}$ as described in equation 2, is presented in Figure 3 between *z* = 4 and 50 mm. The simulated values (black crosses) are fitted to the equation 4 (red line).

(4) $$k_{Q_{\mathrm{msr}},Q_{\mathrm{ref}}}^{f_{\mathrm{msr}},f_{\mathrm{ref}}}\left(z\right)=a\cdot \exp\left(b\cdot z\right)+c\cdot \exp\left(d\cdot z\right)$$

with the fitting parameters given in Table 2.

**TABLE 2:** Fitting parameters for $k_{Q_{msr},Q_{ref}}^{f_{msr},f_{ref}}$ as function of depth.

Parameter	Value	Uncertainty
a	0.4402	0.1398
b	−0.4847	0.0221
c	0.9955	0.0015
d	1.900 *10^−5^	3.601 *10^−5^

At measurement depth < 15 mm, $k_{Q_{\mathrm{msr}},Q_{\mathrm{ref}}}^{f_{\mathrm{msr}},f_{\mathrm{ref}}}\left(z\right)$ exhibits a depth dependence, where its magnitude increases with decreasing *z*. At the *d*_max_ of 7 mm, the value of $k_{Q_{\mathrm{msr}},Q_{\mathrm{ref}}}^{f_{\mathrm{msr}},f_{\mathrm{ref}}}\left(7\;\mathrm{mm}\right)$ is larger than unity with a value of 1.0104 ± 0.0072. At larger depths > 15 mm, $k_{Q_{\mathrm{msr}},Q_{\mathrm{ref}}}^{f_{\mathrm{msr}},f_{\mathrm{ref}}}\left(>15\;\mathrm{mm}\right)$ varies within +‐ 0.2% and can be regarded as constant with a value of 0.9964 ± 0.0025.

### 3.2 Chamber's perturbation correction factors

**FIGURE 4:**
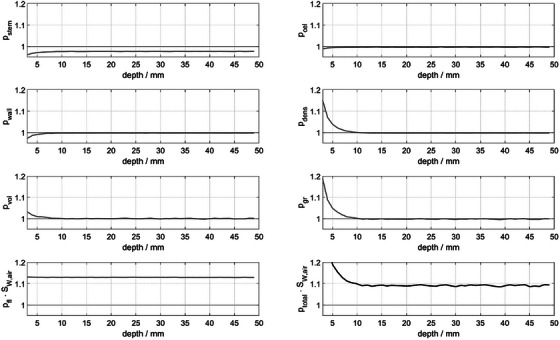
Chamber's perturbation correction factors of the Semiflex 3D ionization chamber as a function of measurement depth *z*.

At depths < 15 mm, all perturbation correction factors, except for *P*_fl_ ⋅ *S*_w,air_, show depth‐dependent behavior as demonstrated in Figure 4. While *P*_stem,_ *P*_cel_ and *P*_wall_ decrease with decreasing depth, both the volume‐averaging *P*_vol_ and density *P*_dens_ perturbation correction factors, and consequently their product *P*_gr _= *P*_vol_ ⋅ *P*_dens_ increase sharply with decreasing measurement depth. At *d*_max,_ *P*_gr_ amounts to 1.018 ± 0.002. Therefore, the resulting $f_{Q_{\mathrm{msr}}}\;\left(z\right)=S_{w,\mathrm{air}}\;\left(z\right)\cdot \prod P_{i}\left(z\right)$ also shows a trend dominated by the depth dependence of *P*_gr._ The perturbation factor data for *d*_max_ as well as depths of 15 mm and 30 mm are presented in Table 3.

**TABLE 3:** Perturbation factors of the Semiflex3D in 7 mm (d_max_), 15 mm and 30 mm depth.

Perturbation factor	7 mm	15 mm	30 mm
*P*_stem_	0.9740 ± 0.0019	0.9757 ± 0.0019	0.9764 ± 0.0019
*P*_cel_	0.9951 ± 0.0020	0.9960 ± 0.0020	0.9962 ± 0.0020
*P*_wall_	0.9961 ± 0.0020	0.9973 ± 0.0020	0.9974 ± 0.0020
*P_gr_*	1.0176 ± 0.0020	0.9995 ± 0.0020	0.9985 ± 0.0020
*P*_dens_	1.0124 ± 0.0020	0.9978 ± 0.0020	0.9975 ± 0.0020
*P*_vol_	1.0051 ± 0.0020	1.0018 ± 0.0020	1.0010 ± 0.0020
*P*_fl_⋅*S*_w,air_	1.1284 ± 0.0023	1.1290 ± 0.0023	1.1288 ± 0.0023

## 4 DISCUSSION

The beam quality correction factor $k_{Q_{\mathrm{msr}},Q_{\mathrm{ref}}}^{f_{\mathrm{msr}},f_{\mathrm{ref}}}\left(z\right)$ for the 3 MV photon beam of the ZAP‐X system is depth‐dependent at measurement depths smaller than 15 mm. As shown in Figure 4, this dependence is strongly influenced by *P*_gr_, which can be expressed as the product of *P*_dens_ and *P*_vol_, whereas the former is shown to be more dominant. In other words, the displacement of a water volume by the low‐density air volume causes the strongest perturbation, an effect also referred to as replacement and displacement perturbation in the literature. The magnitude of the gradient perturbation depends on the dose gradient along the depth axis by assuming a zero dose gradient in the plane perpendicular to the beam's axis within and in the close vicinity of the chamber's dimensions. As a result, the magnitude of *P*_gr_ is larger than unity in the build‐up region, whereas its magnitude approaches a value slightly less than unity in the dose fall‐off region as observed in Figure 4. A similar behavior of other cylindrical chambers has been reported by^11^ for 6 MV photon beam. However, the behavior associated with the 3 MV photon beam reported here is stronger due to a steeper dose gradient along the depth axis.

For reference dosimetry at *d*_max_ = 7 mm as commonly performed, the required $k_{Q_{\mathrm{msr}},Q_{\mathrm{ref}}}^{f_{\mathrm{msr}},f_{\mathrm{ref}}}\left(7\;\mathrm{mm}\right)$= 1.0104 ± 0.0072. Therefore, the estimated error by assuming $k_{Q_{\mathrm{msr}}}$ = 1 due to the current lack of data is also about 1% at this depth. Furthermore, a positioning uncertainty of 0.5 mm here would also lead to a change of $k_{Q_{\mathrm{msr}},Q_{\mathrm{ref}}}^{f_{\mathrm{msr}},f_{\mathrm{ref}}}$ by 0.5%, that is, $k_{Q_{\mathrm{msr}},Q_{\mathrm{ref}}}^{f_{\mathrm{msr}},f_{\mathrm{ref}}}\;\left(6.5\;\mathrm{mm}\right)=1.015$ and $k_{Q_{\mathrm{msr}},Q_{\mathrm{ref}}}^{f_{\mathrm{msr}},f_{\mathrm{ref}}}\;\left(7.5\;\mathrm{mm}\right)=1.0076.$ Therefore, it is suggested to perform the reference dosimetry at depths > 15 mm using 
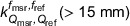
 = 0.9964 ± 0.0025 with practically no depth dependence to minimize the associated uncertainty. It is noteworthy that our simulation model does not consider electron contaminations. Therefore, the reported values in the build‐up region are also associated with higher uncertainty. This limitation does not apply to larger depths beyond *d*_max_. Furthermore, the gradient perturbation depends strongly on the radius of the air cavity. The Semiflex 3D chamber used in this study has an inner radius of 2.4 mm. For larger chambers, the reported depth dependence of the perturbation factor and consequently the beam quality correction factor is expected to be stronger.

## 5 CONCLUSION

The beam quality correction factor $k_{Q_{\mathrm{msr}},Q_{\mathrm{ref}}}^{f_{\mathrm{msr}},f_{\mathrm{ref}}}$ for reference dosimetry at the ZAP‐X using the 25 mm diameter reference cone and the PTW Semiflex 3D chamber was studied for measurement depths between 4 and 50 mm. At the depth of dose maximum *d*_max_ = 7 mm $\;k_{Q_{\mathrm{msr}},Q_{\mathrm{ref}}}^{f_{\mathrm{msr}},f_{\mathrm{ref}}}$ was found to be larger than unity, mainly attributed to the strong depth dependence of the gradient perturbation correction factor. Based on these findings, it is therefore a better practice to perform the reference dosimetry at the ZAP‐X system at depths larger than 15 mm (up to the investigated 50 mm) to minimize the associated uncertainty due to chamber's positioning, where the $k_{Q_{\mathrm{msr}},Q_{\mathrm{ref}}}^{f_{\mathrm{msr}},f_{\mathrm{ref}}}$ can be considered as depth‐independent. For these depths, a $k_{Q_{\mathrm{msr}},Q_{\mathrm{ref}}}^{f_{\mathrm{msr}},f_{\mathrm{ref}}}$ value of 0.9964 ± 0.0025 can be used.

## AUTHOR CONTRIBUTIONS

Katrin Saße performed all measurements, contributed to the simulations, and wrote the manuscript. Karina Albers contributed to the measurements, performed the Monte Carlo simulations, and evaluations of the simulation results. Daniela Poppinga contributed to the evaluation of the results and writing the manuscript. Georg Weidlich contributed to the scientific discussions and review of the manuscript. Björn Poppe contributed to the scientific discussions and review of the manuscript. Hui Khee Looe contributed to the conception of the work, the Monte Cao simulations, and the writing of the manuscript.

## CONFLICT OF INTEREST STATEMENT

Daniela Poppinga is employee of PTW Freiburg. Georg Weidlich is employee of ZAP Surgical.
